# The Impact of Social Network Characteristics on Health among Community-Dwelling Older Adults in Korea: Application of Social Network Analysis

**DOI:** 10.3390/ijerph19074013

**Published:** 2022-03-28

**Authors:** Byoung-Jin Jeon, Kang-Hyun Park

**Affiliations:** 1 Department of Occupational Therapy, Kangwon National University, Samcheok-si 25947, Korea; nomadot@daum.net; 2 Department of Occupational Therapy, Baekseok University, Cheonan-si 31065, Korea

**Keywords:** community-dwelling older adults, friend network, social network analysis, health

## Abstract

Objectives: Population aging is a reality in most developed countries. In this era, an important health issue for these countries is promoting health and quality of life in the elderly population. Older adults’ social networks are associated with health and quality of life. Therefore, this study examines the association between the characteristics of social networks (friendship networks) and the subjective well-being of community-dwelling older adults. Methods: This study was conducted between June 2015 and August 2015 with a total of 146 participants. The size and density of social networks were analyzed using social network analysis. Additionally, to investigate the impact of social networks on health, a multiple linear regression analysis was performed using NetMiner 4.0. Statistical significance was set at *p* < 0.05. Results: In terms of Model 1, which used social network characteristics as variables, the higher the out-degree (376.161) and in-closeness (201.825), the better the health. In contrast, the higher the in-degree (−279.167) and out-closeness (−52.620), the poorer the health. Regarding Model 2, which used sociodemographic characteristics as variables, the higher the out-degree (218.747) and in-closeness (170.075), the better the health. In addition, religion had a negative effect on health, and a high level of education had a positive effect on health. Conclusions: The findings suggest that higher out-degree and in-closeness intensity positively affect the health of older adults, but higher in-degree and out-closeness intensity negatively affect health. Therefore, health professionals should use appropriate strategies to increase the strength of social networks to improve the health of older adults living in the community.

## 1. Introduction

Social networks can comprise different types of social contacts, including close friends, family members, and neighbors, as well as peripheral contacts [1]. Having social relationships is considered an important source of mental and physical health [2,3]. For instance, Cohen (1988) [4] suggested that social support from an individual’s social network may have a primary effect on well-being. Other public and gerontological research has identified social network characteristics as key elements of successful aging [5,6].

For older adults, having a network of friends and socializing are crucial sources of support for health and well-being [7,8]. Furthermore, according to previous literature, interaction with friends or others tends to affect the individual’s satisfaction over their lifetime [9,10,11,12,13,14]. In fact, multiple disciplines, including medical sciences, psychology, sociology, and economics, have illustrated that a larger social network and relationships lead to greater life satisfaction and well-being among older adults [14,15]. Older adults who are embedded in social networks that are characterized by greater social capital report higher well-being, with less loneliness and anxiety, and greater happiness [11]. Similarly, other research has demonstrated that elderly people with a larger social network are happier [9,16], and have higher levels of well-being [11,12,14,17].

However, due to changes in social roles and the decline in the physiological functioning of older adults, their social networks are relatively small and become smaller as they age [18,19]. With an increase in age, their physical functions gradually decline and they eventually face death. Based on the convoy model, older adults will follow fewer fellow travelers on the road of life [20]. Research has suggested that, although the size of social networks among older adults is large, the core of close contacts may remain stable, regardless of changes caused by aging [20].

When considered together, the socioemotional selectivity theory [21] and the convoy model [20] suggest that older adults may deliberately restrict their social relationships to only emotionally-close social friends to benefit their well-being. Therefore, we can predict that the overall social network size will decrease with age, without reducing emotionally-close relationships that are relevant to well-being [22]. Similarly, other research has pointed out that the impact of social networks on the well-being of older adults depends on the network structure and quality of interpersonal relationships [12,13].

Despite comprehensive research on the association between social networks and subjective well-being and health, several aspects of this relationship remain unclear [22]. There is a number of studies on the role of social networks in the life of older adults [23,24,25]. However, research has tended to identify older adults’ networks based on the type or quantity of the relationships [26,27]. For example, some studies only investigated the influence of time spent with friends or frequency of contact on subjective well-being [28,29,30]. Therefore, instruments and questionnaires available to measure friendship networks among older adults are limited [31]. To fill this gap, research that uses scientific analysis, namely social network analysis (SNA), is required to provide a more complete overview of the effect of friend networks on subjective well-being by analyzing various aspects of social networks, including in- and out-degree distribution.

Social network analysis (SNA) is an optimal research tool for examining peer dynamics because it maps out peer-dyadic interdependencies in a larger, social-group context [32]. In addition, by conducting SNA, researchers can illustrate patterns of structural connectivity using a visual analysis of the network by generating a statistical description [33]. Therefore, SNA can help researchers analyze friendship networks and identify the characteristics of friendship ties that can influence behaviors. Several studies have demonstrated that SNA is a useful method for assessing both the structure of peer relationships and friendship networks [34,35].

Based on the previous literature, we propose the following hypotheses. First, individuals have different social network characteristics. Second, individual’s social network characteristics are associated with subjective health. Therefore, the purpose of this study is to examine the association between friendship network characteristics and subjective well-being in community-dwelling older adults.

## 2. Materials and Methods

### 2.1. Participants and Processing

This study included community-dwelling older adults in South Korea with the following inclusion criteria: (1) community-dwelling adults over 60 years of age, (2) who have lived in South Korea for at least one year, and (3) are without any cognitive impairments. The participants were recruited from a local senior center using convenience and snowball sampling methods. The reason the study subjects were recruited from one senior center was that information had to be obtained through a chain of people. To use SNA, it is necessary to obtain and confirm information through a chain of people, originating from respondents 1 and 2, and progressing through the people they point out [36]. Accordingly, to confirm the interactions among the participants, they had to know each other’s names; therefore, recruitment of the participants for the study was limited to one senior center.

The research team provided information regarding the aim of the study and obtained written informed consent from all participants before starting the study. This study was conducted between June 2015 and August 2015, with a total of 146 respondents. It was approved by the Institutional Review Board of Kangwon National University (KWNUIRB-2015-05-002-001).

### 2.2. Instruments

#### 2.2.1. Health

To measure the participants’ health status, there were two questions. The first question asked about self-rated health. Self-rated health was measured by asking the question “In general, how would you rate your health?” with the response options of “excellent, very good, good, fair, or poor”, where higher scores indicated a better subjective health status [37]. Since the 1950s, self-rated health has been one of the most frequently used variables in gerontological and health research [37]. While self-rated health is a subjective, general indicator, several community-based cohort studies have demonstrated a relationship between responses to a question assessing self-rated health and mortality [38,39,40,41,42,43]. The second question asked about the participants’ objective health status. The participants were asked how many times they had visited a medical institution in the past year.

#### 2.2.2. Social Network

In the SNA framework, the participants and their friends indicated their friendships by providing a list of their friends. Friendships were then represented by social tie connections on a graph. Individuals were directly linked to their friends and indirectly to the friends of their friends, forming a large social network of relationships. In this study, to measure the social network of the participants within the group, the participants were asked to write a maximum of three names of people in the senior center that they wanted to perform hobbies with. This is a simple sociometric assessment strategy developed by Moreno (1934) [44].

### 2.3. Data Analyses

Descriptive analyses were conducted to understand the means and standard deviations of all variables using SPSS version 22 (IBM, Armonk, NY, USA). The size and density of the social networks were analyzed using NetMiner 4.0 version (Cyram, Seongnam-si, South Korea). Additionally, to investigate the impact of social networks on health, a multiple linear regression analysis was performed using NetMiner 4.0. Statistical significance was set at *p* < 0.05.

## 3. Results

### 3.1. Characteristics of the Study Population

The sociodemographic characteristics of the participants are described in Table 1, which shows the demographic characteristics of 146 people who responded to this study. The average age of a total of 146 persons was 75.31 (SD = 7.25), and only 35 (23.97%) men were included. Only five persons (3.42%) graduated from high school and most of the participants (54.80%) did not have formal education.

### 3.2. Characteristics of Health

The health of the participants in this study is illustrated in Table 2. Regarding self-rated health, the average score was 2.36 and most of the participants perceived their health as poor. In addition, the participants reported that they had visited a hospital 12.07 times on average over the past year.

### 3.3. Characteristics of the Identified Social Network

The social network characteristics among the participants are shown in Table 3. In terms of the characteristics of the social network, the average in-degree and out-degree was 1.42 and 4.60, respectively. Additionally, the average size of the social network was 5.62, and the average density was 0.10. Regarding the centrality of social networks, it was found that the average in-degree and out-degree centrality was 0.009 and 0.029, respectively. Further, the average in-closeness and out-closeness was 0.017 and 0.064, respectively.

### 3.4. Association between Social Network Characteristics and Health

To examine the impact of social networks on the health of community-dwelling older adults, a multiple linear regression was conducted using Models 1 and 2, which used social network characteristics and sociodemographic variables, respectively (Table 4). Statistical significance was set at *p* < 0.05. Model 1 used the centrality variable of social networks to analyze causality on health. As a result, F (4.811, *p* < 0.01) was statistically significant and Model 1 was deemed suitable. Additionally, the R^2^ value was 0.324, indicating that the explanatory power was 32.5%. It was demonstrated that the higher the out-degree (376.161) and in-closeness (201.825), the better the health. However, the higher the in-degree (−279.167) and out-closeness (−52.620), the poorer the health.

In Model 2, sociodemographic variables were used as control variables. As a result, F (5.666, *p* < 0.01) was statistically significant, indicating that Model 2 was appropriate. Further, the value of R^2^ was 0.557, indicating that the explanatory power was 55.7%. It was found that the higher the out-degree (218.747) and in-closeness (170.075), the better the health. However, the higher the in-degree (−203.223) and out-closeness (−45.084), the poorer the health. In addition, it was shown that religion had a negative effect on health, and older adults with a high level of education exhibited better health.

## 4. Discussion

The objective of this study was to evaluate the impact of social networks on the subjective health of community-dwelling older adults. The discussion will mainly focus on the research methodology and results. First, in terms of the methodology, previous research on the social networks of older adults tended to focus on whether they are socially connected or not. Although this methodology has the advantage of being clear and easily measurable among social networks of various dimensions [45], it has limitations in that it only measures a cross-section of social networks. To analyze the social network of older adults, various factors, including changes in physical and mental health as well as socio-economic context, should be considered. To narrow the gap in previous research, social network analysis, which is based on the middle range theory, has been conducted in recent research [31,46]. Therefore, the strength of this study is that it describes the factors affecting health in detail by confirming the effects of a strong social network on older adults in South Korea.

Moreover, extant literature regarding the social aspect of health focused on the relationship between social capital and health in older adults [47]. However, the concept of social capital is difficult to define clearly; even when researchers have tried to measure social capital in an empirical context, it is controversial because it is not objectively defined as a general scientific concept. In many studies, social capital was confused with the concepts of “social networks” or “social support.” In other studies, social capital was used in relation to social trust. Many empirical studies used “generalized trust” as the main indicator of social capital [47]. Even in this instance, the concept of social capital has been criticized for not acquiring a unique conceptual status as a scientific term because it simply brings together different factors. To overcome this conceptual limitation, SNA was used in the present study. By using SNA in this study, each participant was connected to their friends to form a single network for analysis. This method is considered an objective approach, as opposed to previous research [31,46,48]. The results showed that the higher the out-degree and in-closeness intensity, the more positive the effect on health, and the higher the out-closeness intensity, the more negative the effect on health. This finding supports previous research that shows that social networks, and the social support delivered through these networks, have a strong effect on the mental and physical health of older adults [31,49].

Clearly, high out-degree intensity indicates that, for extroverted individuals, the centrality of the social network has a large amount of external activity. This implies that despite their age, older people are still active because of their relationships with the outside world. In a study on the relationship between social network type and the well-being of rural older adults, it was found that subjective health status was highest in the extroverted type, who had a large social network composed mainly of non-relatives, whom they are generally close to and meet with frequently [50]. This extrovert group was found to have high levels of cognitive function and life satisfaction as well as low levels of depression, as they maintained relationships with various people and participated in society. However, high in-degree intensity indicates that many people approach the individual because they are popular; this suggests that the strength of a passive network where people from outside come in is high, rather than maintaining social relationships. In other words, actively creating social networks and passively creating social networks affect health in completely different ways. This finding reflects the idea that older adults should actively participate in social activities in the way they want. It was demonstrated that participating in a social activity program developed by experts has a negative effect on the health of older adults. Hence, to promote the health of older adults, public health services should be provided in a way that can increase the out-degree. Based on the results of the study, health experts who work with the elderly or the community, including occupational therapists, physical therapists, and social workers, should recognize the importance of participation in voluntary social activities when developing and promoting health strategies.

Additionally, the high intensity of in-closeness shows that valuable information that affects one’s life is gathered from outside, which implies that older adults receive a lot of help from people around them. This result is consistent with that of Choi et al. (2008) [45]. According to the study, the middle-aged elderly who had retired had adverse health effects as the mechanism of receiving various information from friends was cut off. In terms of older adults living alone, they lose people, such as spouses, children, and friends who had provided advice regarding a healthy lifestyle; thus, their health status is easily affected negatively. Therefore, the strength of in-closeness is the ability to maintain health by continuously receiving health-related information from the outside. This finding is consistent with previous studies showing that social supporters are healthy in many cases. The fact that many social supporters help older adults in their lives is a crucial factor in maintaining their health. In contrast, a characteristic of out-closeness is providing information to other people that will be helpful. It can be interpreted as reaching out to help through various activities outside. This implies that, although the energy and enthusiasm for life gradually diminishes for older adults, they remain in a position to serve someone. This is considered a group that works continuously. This group comprises people in a low socioeconomic position. It is important that public health services and policies analyze the socioenvironmental factors surrounding older adults and change the environment so they can receive social support.

The analysis of the effects of demographic variables revealed that people who have no religion were healthier than people with religion. It is presumed that this finding is contrary to previous research because the participants were recruited from only one local senior center. Additionally, it was shown that the higher the education level, the healthier the individual. According to existing studies, education level is positively associated with health. Finally, the two hypotheses which were established in the study were identified.

However, there are several limitations to the present study. First, among the various characteristics of social networks, this study only considered a friend network without a network of close family or relatives. Thus, it is difficult to closely analyze the various social networks of older adults. Second, as this study only used SNA, there were limitations in gathering qualitative information regarding the friendship network of the participants. Therefore, in future studies, a mixed-method approach should be used to examine the friendship network in-depth [51]. Third, because the study was conducted in 2015, the results should be interpreted carefully. In order to bridge the gap, future study should be performed to compare the differences the characteristics of friend networks over time. Fourth, in this study, we examined the impact of social networks on the subjective health of community-dwelling older adults. Thus, there was limited information regarding objective health, such as symptoms, disabilities, and medication use. Subjective health is widely recognized to be a vital global measure of health status and it has been shown to be a powerful predictor of mortality [52]. However, in order to examine the association between social network and health, both objective and subjective health should be measured. Therefore, future studies should consider measurement of objective and subjective health of the participants. Finally, the sample for the current study was recruited from one specific region, which makes it difficult to generalize. Future research should be conducted through colony sampling at a national level.

## 5. Conclusions

The findings suggest that higher out-degree and in-closeness intensity positively affect the health of older adults; however, higher in-degree and out-closeness intensity negatively affect health. Therefore, health professionals should use appropriate strategies to increase the strength of social networks to improve the health of the elderly living in the community. It is necessary to increase the out-degree and in-closeness, and decrease the in-degree and out-closeness. Based on the results of the current study, we were able to confirm the possibility of applying social network strength as a tool to inform public health intervention for increasing health among community-dwelling older adults.

## Figures and Tables

**Table 1:** Demographic characteristics of the sample (*N* = 146).

Sociodemographic Variables	Total Sample; *N* = 146
Age: mean (SD)	75.31 (7.25)
Sex: *n* (%)	
Male	35 (23.97)
Female	111 (76.03)
Educational attainment: *n* (%)	
Elementary school	42 (28.77)
Middle school	19 (13.01)
High school	5 (3.42)
College degree	0 (0.00)
Other	80 (54.80)

**Table 2:** Characteristics of health in the sample (*N* = 146).

Characteristics of Health	Min	Max	Mean	SD
Self-rated health	1 (very poor)	5 (excellent)	2.36	1.48
Number of hospital visits per year	0	30	12.07	8.425

**Table 3:** Social network characteristics.

Social Network Characteristics		Min	Max	Mean	SD
Characteristics	In-degree	0	6	1.420	1.617
	Out-degree	0	6	4.600	1.195
	Size	0	10	5.620	1.898
	Density	0	1	0.103	0.168
Centrality	In-degree	0	0.038	0.009	0.010
	Out-degree	0	0.038	0.029	0.007
	Size	0	0.050	0.017	0.017
	Density	0	0.131	0.064	0.033

Squares = women who have high centrality; Triangles = men who have high centrality.

**Table 4:** Social network characteristics and sociodemographic variables.

Variables		Model 1			Model 2		
		B	t	Sig	B	t	Sig
Centrality	In-degree	−279.167	−2.039	0.048	−203.233	−1.663	0.105
	Out-degree	376.161	3.619	0.001	218.747	2.099	0.043
	In-closeness	201.825	2.562	0.014	170.075	2.357	0.024
	Out-closeness	−52.650	−2.145	0.038	−45.084	−1.975	0.056
Sociodemographic variables	Sex				1.067	0.684	0.499
	Age				−0.165	−1.825	0.076
	Religion				−3.829	−2.676	0.011
	Education level				0.440	2.409	0.021
Constant		12.687	4.546	0.000	29.903	3.763	0.001
F		4.811 **			5.666 ***		
R^2^		0.325			0.557		

** *p* < 0.01, *** *p* < 0.001.
